# Five-year evaluation of bone health in liver transplant patients: developing a risk score for predicting bone fragility progression beyond the first year

**DOI:** 10.3389/fendo.2025.1467825

**Published:** 2025-02-20

**Authors:** Ejigayehu G. Abate, Amanda McKenna, Liu Yang, Colleen T. Ball, Ann E. Kearns

**Affiliations:** ^1^ Division of Endocrinology, Mayo Clinic, Jacksonville, FL, United States; ^2^ Department of Transplantation, Mayo Clinic, Jacksonville, FL, United States; ^3^ Division of Clinical Trials and Biostatistics, Mayo Clinic, Jacksonville, FL, United States; ^4^ Division of Endocrinology, Diabetes, Metabolism and Nutrition, Department of Medicine, Mayo Clinic, Rochester, MN, United States

**Keywords:** liver transplant, transplant related bone disease, osteoporosis, bone risk factors, fractures, post liver transplant related bone loss, glucocorticoid induced osteoporosis

## Abstract

**Introduction:**

Liver transplant (LT) recipients have a substantial risk of bone loss and fracture. An individual’s risk is highest before and within the first year after transplantation and returns to baseline in some patients but not all. We aim to identify risk factors for bone loss and fracture beyond the first year LT and to create a risk-scoring tool to aid clinicians in identifying those at high risk for bone loss and fracture.

**Methods:**

We conducted a retrospective review of 264 liver transplant recipients between 2011 and 2014, who were followed in our transplant clinic for an additional five years. Clinical records were evaluated at the one-year post-LT visit and subsequently on an annual basis for up to five years.

**Results:**

Over a median follow-up of 3.6 years post-liver transplantation, 40 out of 264 patients experienced disease progression, defined as worsening bone mineral density (BMD), initiation of osteoporosis treatment, or a new fracture. Factors associated with BMD progression included female sex, Caucasian race, new fractures, number of acute rejection events requiring treatment, and lower dual energy X-ray absorptiometry (DXA) scores after the first year post-LT. A risk model was developed using multivariable analysis, with a risk score based on BMD categories. The concordance index was 0.771, indicating good discrimination between those who progressed and those who did not. Risk categories were defined as low (0-4 points), medium (5 points), and high (6-9 points) based on model coefficients. The probability of progression-free survival at two years post-LT was 96.7% for low-risk, 83.1% for medium-risk, and 59.1% for high-risk groups.

**Conclusion:**

We developed a simple, clinically applicable risk score that predicts bone disease progression beyond the first year after LT. This tool may help guide appropriate bone health follow-up, although prospective validation is necessary.

## Introduction

Liver transplantation has been an accepted treatment for end-stage liver disease for over 40 years. Advancements in pre-transplant liver disease management, operative techniques, and reduction in dose and duration of glucocorticoid therapy in most patients after LT have improved longevity. With this improvement in survival, there is a need to understand and manage the longer term consequences of LT to enhance quality of life. Over the past two decades, 40-60% of liver transplant recipients have experienced transplant-related bone disorders, a prevalence that has remained unchanged despite advances in transplant care (1–4).

Significant bone loss and fracture, occurring in 13-56% f cases, are predominantly observed in the period before liver transplantation (pre-LT) and within the first-year post-transplant post-LT, with prevalence rates of 13-56% and 14-60% respectively (3–5). The cause is multifactorial, including excess alcohol use, malnutrition, sarcopenia, cholestatic liver disease, hyperbilirubinemia, hyponatremia, vitamin D deficiency, and hypogonadism which tend to improve after transplantation. Additional variables affecting post-LT bone loss include exposure to high-dose glucocorticoid (GC) within the first few months of transplant for immunosuppression, and reduced mobility due to the impact of GC on muscle and bone, to mention a few (6–8). For this reason, Liver Society practice guidelines, American association for study of Liver diseases and American Society for transplantation, and the European clinical practice guidelines include bone densitometry in all patients undergoing LT evaluation (9, 10).

The long-term impact of liver transplantation on bone health remains inadequately understood. While some studies suggest that bone density may stabilize or improve after the first-year post-transplant in most individuals, this recovery is inconsistent (8–10). A subset of patients continues to experience persistent bone loss and an increased risk of fractures. Bone loss progression is commonly assessed through dual-energy X-ray absorptiometry (DXA), while fracture risk is evaluated using radiographic imaging and clinical diagnosis. However, the mechanisms driving these varied outcomes are unclear and require further investigation to enhance our understanding and management of bone health in LT patients beyond the first year post-LT.

Bone histomorphometry, a technique for assessing bone micro-architecture, demonstrates uncoupling of bone remodeling both before and shortly after liver transplantation, marked by decreased bone formation and increased bone resorption (11, 12). Additional studies suggest that bisphosphonates, which reduce bone resorption, can mitigate early post-transplant bone loss. However, while short-term benefits are evident, long-term data remain limited (13, 14). Although bone remodeling often normalizes within four months, some patients continue to face elevated fracture risk, and no clear guidelines exist for monitoring beyond the first year. Furthermore, current fracture assessment tools, including DXA, have limitations in this population (2–4). Our study aims to identify clinical characteristics and BMD values at one-year post-LT that predict progression of bone disease in five years. Based on our findings, we developed a scoring system to identify patients requiring closer monitoring through clinical screening and bone density assessments.

## Materials and methods

### Study design and participants

**Figure 1:**
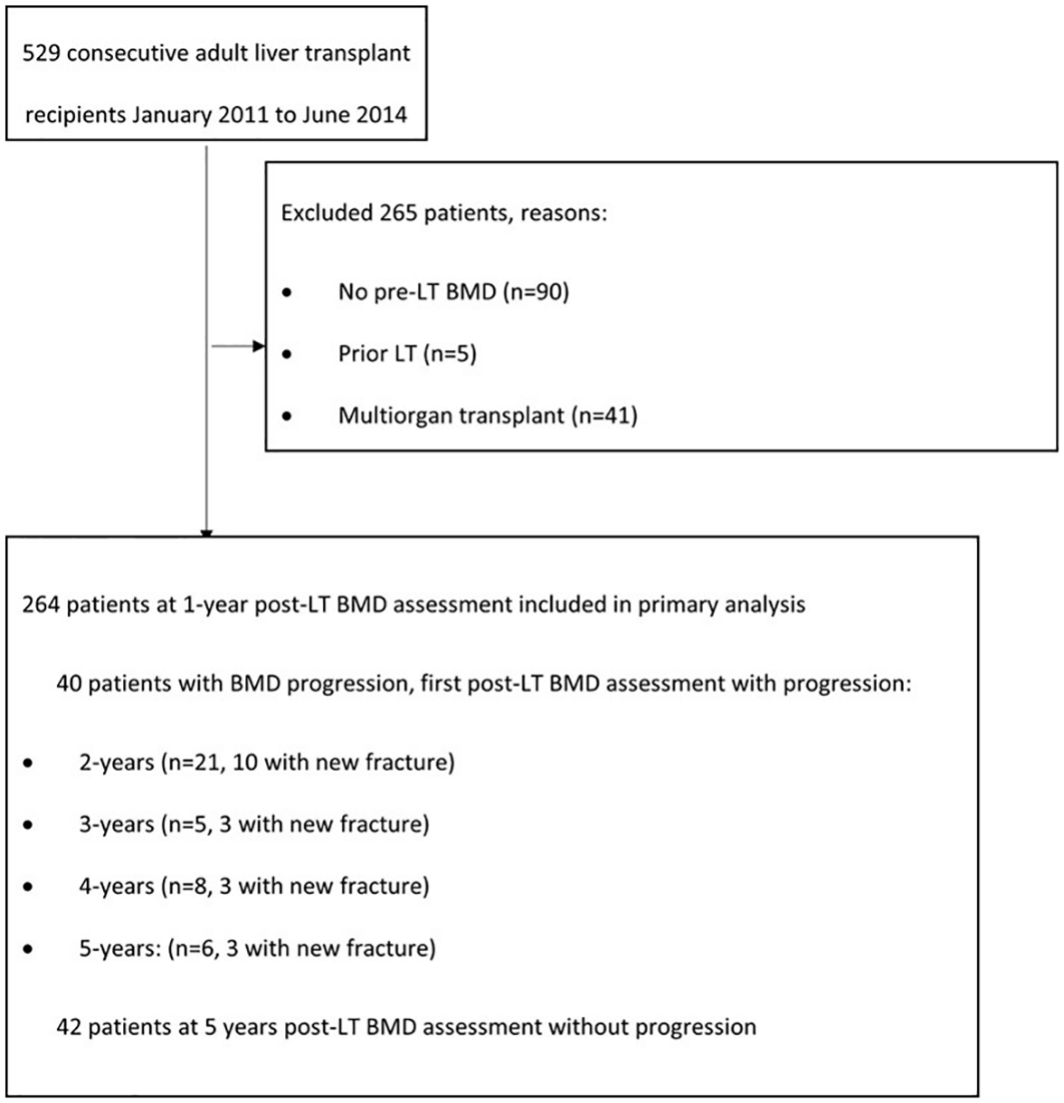
Patient flow diagram.

The study was approved by the institution review board. The cohort included all adult LT recipients from January 2011 through June 2014 who had bone mineral density performed at 1 year post transplant visit. Exclusion criteria were a prior transplant, multiorgan transplant, lack of BMD test results within 1-year post-LT, death within 1 year post transplant, or receiving medication for osteoporosis (**Figure 1**).

Data on biochemistry, the model of end stage liver disease (MELD), bone mineral density (BMD) as measured by DXA result, demography, and clinical endpoints were extracted from participants’ medical records. BMD measured by dual energy x-ray absorptiometry (DXA-GE) are routinely performed at lumbar spine, total hip and femoral neck. Our primary outcome was progression of skeletal fragility after the first-year post LT, defined as transitioning to the subsequent worse BMD diagnosis (osteopenia or osteoporosis), receiving treatment for osteoporosis, or having a new fracture.

### Data collection and BMD data

All patients had DXA scans performed at our facility, and serial comparisons were available. All patients had BMD of the lumbar spine, total hip, and femoral neck measured by DXA using a GE Lunar iDXA (General ElectricCross-calibration of the multiple scanners is routinely performed with a phantom; to provide accurate longitudinal assessment of BMD. BMD results were classified using the World Health Organization diagnostic criteria, defined as osteoporosis if T score is -2.5 or lower, osteopenia if T score is between -1 to -2.4, and normal if T score -1 or higher. Review of electronic medical record documentation including clinical notes and imaging was performed for subjects at one year post LT. Fractures were considered fragility related if occurred from a fall of standing height or from low energy injury reported confirmed in the clinical history. If the cause of the fracture was uncertain, the patient was reported not to have a fracture. We obtained the number of any rejections occurring within the first year of post-LT by review of transplant follow-up notes. Most patients did not have rejection. Bone loss progression was defined as having one or more of the following: Transitioned from normal BMD at 1-year post-LT to osteopenia or osteoporosis, transitioned from osteopenia at 1-year post-LT to osteoporosis, received treatment for osteoporosis after 1-year post-LT, or had a new fracture after 1-year post-LT. BMD classification was based on the lowest BMD T score available for the corresponding clinical visit (**Table 1**).

**Table 1:** Patient characteristics.

	N	n (%) or median (IQR)
Pre-transplant information		
Female sex	264	88 (33.3%)
Race		
Caucasian	264	222 (84.1%)
African American	264	23 (8.7%)
Other	264	14 (5.3%)
Not reported	264	5 (1.9%)
Body mass index (kg/m2)	264	27.8 (24.9, 32.1)
Primary liver disease	264	
Cirrhosis, Type C		88 (33.3%)
Alcoholic cirrhosis		42 (15.9%)
Cirrhosis, fatty liver (Nash)		33 (12.5%)
Cirrhosis, cryptogenic idiopathic		30 (11.4%)
Alcoholic cirrhosis with hepatitis C		17 (6.4%)
Cirrhosis, autoimmune		11 (4.2%)
Primary biliary cirrhosis		10 (3.8%)
Cirrhosis, other		4 (1.5%)
Primary sclerosing cholangitis		12 (4.5%)
Metabolic disease		8 (3.0%)
All other diagnoses		9 (3.4%)
MELD score	264	18 (11, 25)
History of prednisone use	261	15 (5.7%)
Information collected at 1^st^ annual follow-up visit		
Age (years)	264	60 (54, 66)
Fractures	264	
No fractures		204 (77.3%)
Pre-transplant fracture, no new fracture in 1st year post-LT		21 (8.0%)
New fracture in 1st year post-LT with or without history of		39 (14.8%)
pre-transplant fracture		
Total prednisone dose in 1st year post-LT	264	1.10 (1.10, 1.10)
Number of rejections in 1st year post-LT	264	
0		217 (82.2%)
1		35 (13.3%)
2		10 (3.8%)
3		2 (0.8%)
Tacrolimus use in 1st year post-LT	254	232 (91.3%)
Mycophenolate in 1st year post-LT	253	33 (13.0%)
Sirolimus use in 1st year post-LT	264	8 (3.0%)
Lowest BMD T score at 1st annual follow-up	264	-1.60 (-2.20, -0.90)
Spine T score	256	-0.40 (-1.30, 0.40)
Femoral neck T score	263	-1.50 (-2.00, -0.75)
Total hip T score	263	-1.10 (-1.70, -0.30)

### Immunosuppression

The immunosuppressive regimen protocol following LT is mycophenolate mofetil (CellCept) (for 2 months), prednisone (taper completed by 4 months), and tacrolimus indefinitely. If the patient has high-risk hepatocellular carcinoma, mycophenolate mofetil will be stopped as early as day 21 post LT, and the patient will remain only on tacrolimus. If the patient has renal insufficiency, mycophenolate is continued as maintenance therapy along with tacrolimus to reduce tacrolimus levels to avoid further decline in kidney function. Patients with moderate to severe acute cellular rejection receives intravenous (IV) methylprednisolone 1 gram every other day for a total of 3 doses. Patients then undergo repeat liver biopsy, and if the biopsy indicates persistent moderate rejection, treatment with another cycle of IV methylprednisolone is given. Patients receive thymoglobulin if organ rejection persists. Patients receive a higher dose of tacrolimus maintenance therapy for mild acute cellular rejection. The protocol of immunosuppression did not change during the study period.

### Statistical analysis

Associations of patient characteristics with bone loss progression after the 1-year post LT visit were evaluated using Cox proportional hazards regression models, where hazard ratios (HRs) and 95% confidence intervals (CIs) were estimated. Patients without bone loss progression were censored at the last available BMD assessment prior to any graft failure. To create a scoring algorithm that classifies patients based on their risk of bone loss progression after their first annual post-LT BMD assessment, a multivariable Cox proportional hazards regression model was developed using a backward selection approach, with a focus on reduction in the Akaike Information Criterion (AIC). The multivariable Cox proportional hazards regression model was developed by including variables with p ≤ 0.20 from the univariable analysis. Variables were removed one at a time, based on the largest p-value, until no further reduction in the Akaike Information Criterion (AIC) was observed (**Table 2**). The BMD measurement at 1 year post-LT was selected over pre-LT BMD to identify predictors associated with the 1-year post-LT DXA assessment, independent of pre-transplant values, and the closer proximity of the BMD at 1 year Post-LT visit.

**Table 2:** Associations with BMD progression after 1 year post-liver transplant visit.

	N	No. of events	Single variable analysis		Multivariable model	
			HR (95% CI)	P	HR (95% CI)	P
*Pre-transplant information*						
Sex						
Male	176	16	1.00 (reference)		1.00 (reference)	
Female	88	24	2.89 (1.54-5.45)	.001	2.29 (1.20-4.38)	0.012
Race						
African American	23	5	1.00 (reference)		1.00 (reference)	
White/Other/Unknown	241	35	6.37 (0.86-47.11)	.070	6.47 (0.85-49.11)	0.071
Body mass index (-5 kg/m2)			1.23 (0.90-1.70)	.20		
30 kg/m2 or higher	87	11	1.00 (reference)			
< 30 kg/m2	177	29	1.16 (0.58-2.34)			
Primary liver disease						
Cirrhosis, Type C (yes vs. no)	88	11	0.84 (0.42-1.69)	.62		
Alcoholic cirrhosis (yes vs. no)	42	8	1.47 (0.67-3.19)	.34		
Cirrhosis, fatty liver (Nash) (yes vs. no)	33	6	1.06 (0.44-2.53)	.90		
Cirrhosis, cryptogenic idiopathic (yes vs. no)	30	7	1.41 (0.62-3.21)	.41		
MELD score (+15)			1.50 (0.87-2.26)	.17		
18 or less	138	18	1.00 (reference)			
More than 18	126	22	1.22 (0.65-2.27)			
Information collected at 1 year post LT visit						
Age (+10 years)			1.26 (0.86-1.83)	.24		
60 years or younger	132	19	1.00 (reference)			
Older than 60 years	132	21	1.04 (0.56-1.94)			
Fractures						
No fractures	204	20	1.00 (reference)		1.00 (reference)	
Pre-transplant fracture, no new fracture in 1st year post-LT	21	5	2.04 (0.76-5.45	.16	2.03 (0.74-5.56)	.17
New fracture in 1st year post-LT with or without history of pre-transplant fracture	39	15	4.94 (2.52-9.68	<.001	4.48 (2.22-9.05)	<.001
Total prednisone dose in 1st year post-LT (+0.5)			1.06 (0.97-1.15)	.18		
1.1 or less	201	24	1.00 (reference)			
More than 1.1	63	16	2.45 (1.30-4.63)			
No. of rejections in 1st year post-LT (+1)			1.76 (1.12-2.76)	.014	1.74 (1.07-2.84)	0.026
0	217	28	1.00 (reference)			
1	35	8	2.25 (1.02-5.00)			
2 or more	12	4	2.71 (0.95-7.74)			
Tacrolimus use in 1st year post-LT (yes vs. no/unk)						
No/Unknown	32	7	1.00 (reference)			
Yes	232	33	0.55 (0.24-1.24)	.15		
Mycophenolate in 1st year post-LT						
No/Unknown	231	36	1.00 (reference)			
Yes	33	4	1.05 (0.37-2.97)	.92		
Sirolimus use in 1st year post-LT						
No/Unknown	256	37	1.00 (reference)			
Yes	8	3	2.52 (0.78-8.21)	.12		
Lowest BMD T score at 1-year post-LT follow-up (-1)	75	11	1.81 (1.24-2.64)	.002	1.69 (1.12-2.55)	0.012
-1.0 or higher		11	1.00 (reference)			
Between -2.5 to and -1.0	153	19	0.95 (0.45-1.99)			
-2.5 or lower	36	10	3.03 (1.28-7.17)			
Spine T score at 1-year post-LT follow-up (-1)			1.30 (1.00-1.69)	.046		
Femoral neck T score at 1-year post-LT follow-up (-1)			1.65 (1.13-2.41)	.010		
Total hip T score at 1-year post-LT follow-up (-1)			1.62 (1.16-2.27)	.004		

BMD, bone mineral density; HR, hazard ratio; CI, confidence interval; LT, liver transplant.

BMD progression was defined as having one or more of the following: transitioned to a worse diagnosis (osteopenia or osteoporosis ) based on the lowest BMD T score, received treatment for osteoporosis, or had a new fracture. Patients were censored at the last available BMD assessment prior to graft failure. The multivariable Cox proportional hazards regression model included variables with P≤0.20 from single variable analysis removing one variable at a time based on the largest P value until there was no longer a reduction in the Akaike Information Criterion. Prior to starting the backward selection procedure, some variables were removed from the model due to high correlation. Prednisone dose was not included due to the correlation with the number of rejections (Spearman correlation = 0.74). The only BMD T-score included in the multivariable model prior to backward selection was the lowest T score at 1-year post-LT follow-up. The concordance index for the multivariable model was 0.771 (95% bootstrap CI 0.696-0.867) (15). For body mass index, MELD score, age at 1 year follow-up, total prednisone dose, number of rejections, and lowest bone mineral density T score, unadjusted HRs and 95% CIs were presented for categorized versions of the variables to ease interpretation, but the continuous versions of the variables were used for calculating P values and for consideration in the multivariable model.

The number in parathesis Indicate the unit increase (+) or decrease (-) in the predictor variable associated with the reported Hazard Ratio. For example, Age (+10 years) signifies that the HR corresponds to a 10-year increase in age.

The risk score model was constructed using factors known at one-year post-LT, with points assigned based on variables with p ≤ 0.20. The scoring system included the following: sex (+1 point if female), race (+2 points if not African American), fracture history (+2 points for new fractures post-transplant or +1 point for pre-transplant fractures), number of rejections (+2 points for ≥2 rejections or +1 point for 1 rejection), and lowest BMD T-score (+2 points if T ≤ -2.5 or +1 point if T > -2.4 and < -1.0) (**Table 3**). The point values for the risk score were determined by rounding each model coefficient to the nearest integer (e.g., female sex had a coefficient of 0.828, rounded to 1; non-African American race had a coefficient of 1.870, rounded to 2). This simplification was done to create an easy-to-use risk scoring system (**Supplementary Table 1**).

**Table 3:** Risk score development for predicting BMD progression after 1 year post liver transplant visit.

Variable in model	Model Coefficient	Points
Sex		
Male	Reference	0
Female	0.828	1
Race		
African American	Reference	0
White race, other race, or unknown race	1.870	2
Fractures		
No fractures	Reference	0
Pre-transplant fracture, no new fracture in 1st year post-LT	0.707	1
New fracture in 1st year post-LT with or without history of pre-transplant fracture	1.430	2
No. of rejections in 1st year post-LT, continuous	0.555	
No. of rejections, categories		
0		0
1		1
2 or more		2
Lowest BMD T score at 1-year post-LT follow-up, continuous	-0.524	
Lowest BMD T score at 1-year post-LT follow-up categories		
-1.0 or higher		0
Between -2.5 to and -1.0		1
-2.5 or lower		2

The number of points for the simplified score were determined by rounding the model coefficient up to the nearest integer. For the number of rejections, only 1 patient had more than 2 rejections so those with 2 or more rejections were combined into the same category. The lowest T-score was categorized based on common clinical diagnostic criteria. The risk score is calculated by summing the number of points with a plausible range of 0 to 9. The concordance index for the score created using the model coefficients and the simplified risk score was 0.771 and 0.761, respectively.

The risk score was calculated by summing the points for the included variables, resulting in a plausible score range of 0-9. To evaluate the discriminatory ability of the risk score in predicting bone loss progression, we estimated the concordance index and corresponding 95% confidence intervals (CIs) using bootstrap methods (**Table 2**). Concordance index is a measure of the model’s ability to discriminate between those who progressed and those who didn’t with consideration of the time-to-event and censoring. The concordance index was 0.76 in our cohort. The risk score was categorized into three groups: low (0-4 points), medium (5 points), and high (6-9 points) risk of skeletal fragility progression.

**Figure 2:**
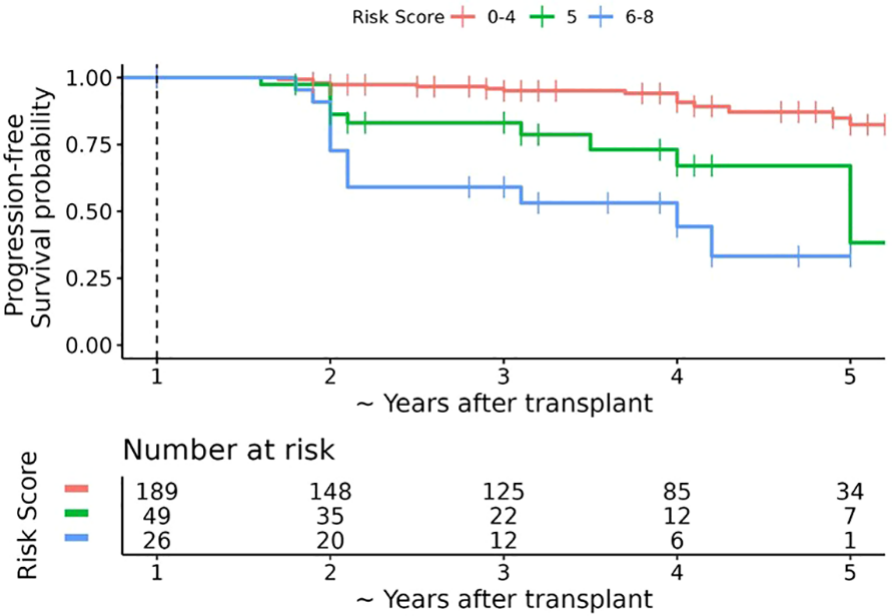
Kaplan-Meier estimates of progression-free survival after 1 year post-transplant bone mineral density (BMD) assessment according to simplified risk score. The dashed vertical line represents the date of the first annual BMD assessment (baseline timepoint). The horizontal axis represents the number of years after the first annual BMD assessment plus 1 year; for ease of interpretation the horizontal axis is labelled as the approximate number of years after transplant. Over a median follow-up of ~ 3.6 years post-transplant (interquartile range ~3 to ~ 5 years post-transplant), 40 patients experienced bone loss progression, 20 of which occurred within 18 months after 1 year post-LT BMD assessment. The overall probability of progression-free survival at ~2.5, ~3.5, and ~4.5 years post-transplant was 90.3% (95% CI 86.3% to 94.4%), 87.1% (95% CI 82.4% to 92.0%), and 78.1% (95% CI 60.0% to 79.6%), respectively.

We assessed the performance of the risk score by plotting Kaplan-Meier estimates of progression-free survival according to risk score categories. Median follow-up time was calculated using the reverse Kaplan-Meier method, with patients who experienced progression censored at the time of progression (**Figure 2**, **Table 4**). All analyses were conducted using R version 4.0.3 (R Foundation for Statistical Computing, Vienna, Austria).

**Table 4:** Kaplan-meier estimates of the probability (%) of progression-free survival after the 1 year post liver transplant visit.

Years after 1 year post LT visit	Simplified Risk Score		
	0-4	5	6-8
1.5 (~2.5 years after transplant)	96.7 (93.8-99.6)	83.1 (71.6-96.5)	59.1 (41.7-83.7)
2.5 (~3.5 years after transplant)	95.1 (91.7-98.7)	73.1 (57.9-92.3)	53.2 (35.5-79.7)
3.5 (~4.5 years after transplant)	87.1 (80.3-94.6)	67.0 (50.2-89.5)	33.2 (15.2-72.7)

Progression was defined as having one or more of the following: transitioned to a worse diagnosis (osteopenia or osteoporosis) based on the lowest BMD T score, received treatment for osteoporosis, or had a new fracture.

## Results

Our cohort included 264 patients who underwent LT at our institution, with bone mineral density testing available at their 1-year post LT visit met the criteria for analysis. **Table 1** describes the characteristics of the cohort including bone mineral density results extracted from their medical records. At one year post LT, the median age was 60 years (IQR 54 to 66 years), 88 (33.3%) were female, 47 (17.8%) had one or more rejections within the first year after LT. The cohort consisted of diverse liver disease as the cause for transplant. 39 (14.8%) had a fracture in the first year after LT, and median lowest BMD T score from the spine, femoral neck, or total hip was -1.60 (IQR -2.20 to -0.90). Fractures were predominantly in the spine (thoracic and vertebral spine).

After 1 year LT visit, 40/264 patients experienced bone loss progression over a median follow-up period of 3.6 years post-LT bone density (IQR 1.0 to 3.6 years), with 21 patients progressing by year 2 post LT visit (**Figure 1**). There were 42 patients who completed 5-year post LT follow-up visits without progression. The remaining 182 patients either had graft failure requiring a second liver transplant (N=24) or were lost to follow-up prior to the 5-year post LT visit. Among those who progressed, the majority were within 2 years of LT (n=21) and 10/21 had new fractures which was not evident in the pre or immediate post-transplant period (**Figure 1**). Eight out of the 10 patients had multiple fractures predominantly in the spine. Clinical characteristics of those who progress include female sex which were 2.89 times more likely to progress than male counterparts, Caucasians or those who identified as others were 6 times more likely to progress than African Americans, those with new fracture at 1 year post LT regardless of history of pre transplant fracture were 5 times more likely to progress than those who did not have reported fracture, patients with one or more episodes of organ rejection were over 2 times likely to progress than those who did not have rejection and low hip DXA scan were also likely to progress. Based on this significant data, we identified female sex, Caucasian race, history of fracture before transplant and within 12 months post LT, organ rejection of 2 or more episodes, and low BMD in osteoporosis range as a significant variable to play a role in progression of bone loss.

Due to the limited number of patients who developed bone loss progression and the strong correlations between some of the factors, the use of multivariable analysis to predict bone loss progression was challenging. We considered any variable with a P ≤ 0.20 from single variable analysis in our model, excluding prednisone dose due to its correlation with the number of rejections. The lowest BMD T score at 1-year post-LT was the only BMD measurement considered in the model. The remaining factors under consideration were included in a multivariable Cox proportional hazards regressions model. Using a backward elimination approach, one variable at a time was removed from the model based on the highest P value until there was no longer a reduction in the AIC. The final multivariable model included 5 factors, sex, race, fracture history, number of rejections within one-year post-LT, and the lowest BMD T score at 1-year post-LT (concordance index = 0.771, 95% CI 0.696-0.867).

To create the risk score, each model coefficient was rounded up to the nearest integer and these individual scores were summed to create a score that ranges from 0 to 9 (**Table 3**). After reviewing the observed proportion of patients who developed bone loss progression by the 2-year post LT visit according to the risk score (**Supplementary Table 1**), we combined the risk scores into 3 risk categories: low risk 0-4, medium risk 5, and high risk 6-9. The probability of progression-free survival by the 2-year post LT visit was 96.7% (93.8% to 99.6%), 83.1% (71.6% to 96.5%), and 59.1% (41.7% to 83.7%) for the low, medium, and high-risk categories, respectively (**Table 4**, **Figure 2**).

## Discussion

Our findings shows that certain characteristics identified as risk factors for osteoporosis and fractures in the non-transplant population are also significant for bone health changes in post LT patients. Specifically, female gender, Caucasian race/ethnicity, and a history of previous fractures were identified as important risk factors (14, 16). Refinement in risk assessment for this population comes from our identification of liver transplant (LT)-related factors associated with skeletal health deterioration. Specifically, a higher number of rejection episodes and low bone density in the hip were significant factors.

Our study agrees with previous work that most fractures occur early, before transplant or within the first 1-2 years of transplant (13, 14). In contrast to prior studies, we did not observe any association between the type of liver disease (alcohol, Non-alcoholic liver disease, hepatitis c) and progression of bone disease (14, 17). However, we did not look at those with cholestatic liver disease separately due to our small number of patients in the cohort.

In contrast to prior studies, our study highlights the importance of recognizing the risk factors for bone loss and fracture after the first year of transplant unique to liver transplant recipients. More specifically, similar to the non-transplant population, females at any time were more likely to progress than their male counterparts. Women have lower bone mass at any point compared to men, so it was not surprising to discover that the most significant effect of liver disease on the bone was higher in women than men. Prior studies have questioned the validity of BMD in predicting bone disease in those receiving liver transplants (2). In this study, we found that BMD is essential in the risk stratification of patients at high risk for progression in conjunction with other clinical factors identified in this study. BMD at 1-year post LT was also helpful in predicting those who are likely to progress. We noted that patients with BMD at any site (spine, femoral neck, or total hip) in the osteoporosis range at 1-year post LT were three times more likely to progress compared to those with normal BMD (T score >-1) at any site. In addition, a one standard deviation (SD) decrease in the total hip BMD at 1-year post LT was strongly associated with BMD progress after the first annual post-LT follow-up. A change in BMD by one standard deviation change in the femoral neck and spine was also trending towards a positive prediction of progression, although not significant (**Table 2**).

Furthermore, ethnic variation in peak bone mass is likely to explain the race differences noted in our study rather than the mere impact of transplant alone. African Americans generally have been shown to have a higher bone mineral density at baseline than white Americans, and fracture rate also appears to be lower in AA at any skeletal sites compared to whites in nontransplant patients (16). We are not aware of any known differences in the mechanism of bone loss between AA and Caucasians other than the fact that AA may have higher bone mass at baseline than Caucasians but whether the rate of loss is different between the two groups is unknown. Unique to our transplant cohort, we noticed that although Caucasians had a higher risk for progression than African Americans, a higher proportion in the AA group 5/23 (22%) progressed versus 35/241(14%) Caucasians. The number of African American LT population was low but carried a concerning trend of disease progression raising the possibility that perhaps the rate of bone loss can be higher in AA population and may need close follow-up. Future studies may help understand potential race differences in how organ failure and transplantation affect different ethnic groups.

Glucocorticoid use, mycophenolate, and Tacrolimus use were not associated with progression risk, in keeping with the concept that bone disease in transplant patients is a unique entity caused by multifactorial pathways rather than explained by immunosuppression alone. High glucocorticoid (GC) use is typically limited to the first 4-6 months post-LT and discontinued in most individuals. GC is known to affect the bone by uncoupling bone resorption and bone formation, resulting in increased bone resorption by inhibiting gonadal steroids, increased urinary calcium excretion by inhibiting intestinal and renal calcium reabsorption, and secondary hyperparathyroidism and reduced bone formation by inhibiting type I collagen, osteocalcin, insulin-like growth factors, and bone matrix proteins, receptor activator for nuclear factor kappa B ligand (RANK-L (18, 19). The effect of GC on the bone goes well beyond the withdrawal of GC, and the GC effect on the bone is apparent even in lower doses (19, 20).

Our study shows that frequent organ rejection (>2) episodes rather than GC use may be an important clinical tool that can differentiate those with long-term effects on the bone from those with low rejection episodes. Our study noted that a higher number of rejections (>2 episodes) was associated with a significantly high risk of bone progression. Those with a higher rejection frequency in the first year of transplant may have received a higher dose of steroid than those with less frequent rejection events and are overall sicker. In addition, the finding may be in part explained by the GC effect on various organs, and GC-sparing therapies such as calcineurin inhibitor (Tacrolimus) may have a favorable effect on bone health GC asserts a direct effect on reducing osteoblast replication, differentiation, and lifespan resulting in a decline in bone formation. 33/232 (14%) of patients using Tacrolimus in first-year post-LT progressed, whereas only 4/33 (12%) patients using mycophenolate progressed. Though the findings were insignificant, the trend was that those on Tacrolimus had half the probability of progressing HR 0.55 (0.24-1.24) compared to those not on Tacrolimus. Prior studies have shown that early glucocorticoid withdrawal improves bone mass recovery (21–24). Calcineurin inhibitors (cyclosporine A (CsA) and Tacrolimus) are GC-sparing immunosuppressants that have been instrumental in reducing GC use. The effects of cyclosporine A (CsA) on bone health are unclear, though it generally appears to increase bone resorption and lead to bone loss. In contrast, tacrolimus is associated with less bone loss, likely due to reduced glucocorticoid (GC) use rather than direct effects on bone cells (13, 22). Glucocorticoids, mycophenolate, tacrolimus, and liver disease type were not significantly linked to increased bone disease progression, suggesting that bone disease in transplant patients has unique, multifactorial causes. Pre-transplant fracture notably was associated with BMD progression, with 38% (15 of 39) of patients with pre-transplant fractures experiencing progression—five times higher than those without fractures. Even without new post-transplant fractures, these patients had twice the risk of BMD progression, observed in 5 of 21 patients, emphasizing pre-transplant fractures as a key factor in post-transplant bone disease.

Long-term follow-up of LT patients beyond the first year should include fracture risk assessment through a comprehensive clinical history of known fracture risk factors. Particular attention should be given to patients who have received high doses of glucocorticoids for frequent rejections in the early post-transplant years. Clinicians should make every effort to obtain adequate clinical history and, when in doubt, obtain spine imaging to evaluate for asymptomatic fracture.

Prior studies have shown that the risk of asymptomatic vertebral fracture in pre-LT recipients was as high as 56% (2, 25). The study underscores the importance of pre- or within one year of LT fracture assessment by spine imaging to evaluate for radiographic evidence of fracture or clinical history suggestive of fractures such as height loss and kyphosis.

Currently, there are no standardized guidelines for the optimal interval between bone mineral density (BMD) testing or for the management of patients with liver transplant (LT)-related bone disease. Based on our findings, we propose that patients with a clinical risk score greater than five should be considered for additional spine imaging, such as plain radiographs of thoracic and lumbar spine, to evaluate for asymptomatic fractures, or should be considered for early treatment intervention. Prospective studies are needed to further validate the efficacy of this risk tool in guiding patient selection for treatment.

This study has several limitations, largely due to its retrospective design. First, we did not account for comorbidities or medications that may impact bone density, such as thiazide diuretics for hypertension or conditions such as type 2 diabetes. Additionally, because the study relied on retrospective chart reviews, not all relevant medical information was consistently recorded, particularly for patients managed by external institution (21, 22, 26).

Not unexpectedly, the presence of fracture at any time (pre-LT and within 1-year post-LT) is one of the strongest predictors of 5-year disease progression compared to those that did not fracture. The presence of a new fracture in the first-year post-LT visit, regardless of prior fracture, was highly correlated with disease progression (p <0.001).

Moreover, patients who received treatment for osteoporosis after the first year post-LT were classified as having “bone loss progression,” based on the assumption that treatment initiation reflects a clinical decision prompted by observed bone loss or increased risk. While it is possible that some patients may have started treatment as a preventive measure, we lacked sufficient data to differentiate between those treated for active bone loss and those treated prophylactically. Further studies should aim to clarify this distinction.

## Conclusion

We developed a risk-scoring tool to enable clinicians to identify individuals with the highest risk of deterioration in bone health, defined as time to decline to the subsequent worse diagnosis (osteopenia and osteoporosis) based on the lowest BMD T score, received treatment for osteoporosis or had a new fracture. The BMD progression risk score is an easy-to-calculate scoring system based on information collected at the one-year follow-up assessment after a liver transplant. This tool though an encouraging start, will require prospective validation.

## Data Availability

The raw data supporting the conclusions of this article will be made available by the authors, without undue reservation.

## References

[1] Cohen A Sambrook P Shane E. Management of bone loss after organ transplantation. J Bone Miner Res. (2004) 19:1919–32. doi: 10.1359/jbmr.040912 15537434

[2] Krol CG Dekkers OM Kroon HM Rabelink TJ van Hoek B Hamdy NA. No association between BMD and prevalent vertebral fractures in liver transplant recipients at time of screening before transplantation. J Clin Endocrinol Metab. (2014) 99:3677–85. doi: 10.1210/jc.2014-1469 25057874

[3] Krol CG Dekkers OM Kroon HM Rabelink TJ van Hoek B Hamdy NA. Longitudinal changes in BMD and fracture risk in orthotopic liver transplant recipients not using bone- modifying treatment. J Bone Miner Res. (2014) 29:1763–9. doi: 10.1002/jbmr.2214 24644003

[4] Abate EG Vega MV Rivas AM Meek S Yang L Ball CT. Evaluation of factors associated with fracture and loss of bone mineral density within 1 year after liver transplantation. Endocr Pract. (2021) 27:426–32. doi: 10.1016/j.eprac.2020.10.008 33645516

[5] Butin S Griffoul I Espitalier F Salame E Mulleman D Goupille P. High incidence of vertebral osteoporotic fracture within the first year after liver transplantation. Clin Exp Rheumatol. (2017) 35(6):913–8. 28516878

[6] Chiu YC Liao PS Chou YT Lin CL Hung CH Lin CC. The incidence and risk factors of hip fracture after liver transplantation (LT): A nationwide population-based study. BioMed Res Int. (2019) 2019:5845709. doi: 10.1155/2019/5845709 31950045 PMC6944971

[7] Danford CJ Trivedi HD Bonder A. Bone health in patients with liver diseases. J Clin Densitom. (2020) 23:212–22. doi: 10.1016/j.jocd.2019.01.004 30744928

[8] Bergmann PJ. Change in bone density and reduction in fracture risk: A meta- regression of published trials. J Bone Miner Res. (2019) 34:1976. doi: 10.1002/jbmr.3835 31433513

[9] Martin P DiMartini A Feng S Brown R Jr. Fallon M. Evaluation for liver transplantation in adults: 2013 practice guideline by the American Association for the Study of Liver Diseases and the American Society of Transplantation. Hepatology. (2014) 59:1144–65. doi: 10.1002/hep.26972 24716201

[10] Guichelaar MM Malinchoc M Sibonga J Clarke BL Hay JE. Bone metabolism in advanced cholestatic liver disease: analysis by bone histomorphometry. Hepatology. (2002) 36:895–903. doi: 10.1053/jhep.2002.36357 12297836

[11] Guichelaar MM Malinchoc M Sibonga JD Clarke BL Hay JE. Bone histomorphometric changes after liver transplantation for chronic cholestatic liver disease. J Bone Miner Res. (2003) 18:2190–9. doi: 10.1359/jbmr.2003.18.12.2190 14672354

[12] Vedi S Ninkovic M Garrahan NJ Alexander GJ Compston JE. Effects of a single infusion of pamidronate prior to liver transplantation: a bone histomorphometric study. Transpl Int. (2002) 15:290–5. doi: 10.1111/j.1432-2277.2002.tb00167.x 12072899

[13] Bodingbauer M Wekerle T Pakrah B Roschger P Peck-Radosavljevic M Silberhumer G. Prophylactic bisphosphonate treatment prevents bone fractures after liver transplantation. Am J Transplant. (2007) 7:1763–9. doi: 10.1111/j.1600-6143.2007.01844.x 17511759

[14] Vedi S Greer S Skingle SJ Garrahan NJ Ninkovic M Alexander GA. Mechanism of bone loss after liver transplantation: A histomorphometric analysis. J Bone Miner Res. (1999) 14:281–7. doi: 10.1359/jbmr.1999.14.2.281 9933483

[15] Harrell FE Lee KL Mark DB. Tutorial in Biostatistics: Multivariable prognostic models: issues in developing models, evaluating assumptions and adequacy, and measuring and reducing errors. Stat Med. (1996) 15:361–87. doi: 10.1002/(SICI)1097- 8668867

[16] Looker AC Melton LJ 3rd Borrud LG Shepherd JA. Lumbar spine bone mineral density in US adults: demographic patterns and relationship with femur neck skeletal status. Osteoporos Int. (2012) 23:1351–60. doi: 10.1007/s00198-011-1693-z 21720893

[17] Bouxsein ML Eastell R Lui LY Wu LA de Papp AE Grauer A. Change in bone density and reduction in fracture risk: A meta-regression of published trials. J Bone Miner Res. (2019) 34:632–42. doi: 10.1002/jbmr.3641 30674078

[18] Stein E Ebeling P Shane E. Post-transplantation osteoporosis. Endocrinol Metab Clin North Am. (2007) 36:937–63. doi: 10.1016/j.ecl.2007.07.008 17983930

[19] Buckley L Guyatt G Fink HA Cannon M Grossman J Hansen KE. 2017 American college of rheumatology guideline for the prevention and treatment of glucocorticoid- induced osteoporosis. Arthritis Rheumatol. (2017) 69:1521–37. doi: 10.1002/art.40137 28585373

[20] van Staa TP. The pathogenesis, epidemiology and management of glucocorticoid- induced osteoporosis. Calcif Tissue Int. (2006) 79:129–37. doi: 10.1007/s00223-006-0019-1 16969593

[21] Feller RB McDonald JA Sherbon KJ McCaughan GW. Evidence of continuing bone recovery at a mean of 7 years after liver transplantation. Liver Transpl Surg. (1999) 5:407–13. doi: 10.1002/(ISSN)1527-6473a 10477842

[22] Goffin E Devogelaer JP Lalaoui A Depresseux G De Naeyer P Squifflet JP. Tacrolimus and low-dose steroid immunosuppression preserves bone mass after renal transplantation. Transpl Int. (2002) 15:73–80. doi: 10.1111/j.1432-2277.2002.tb00133.x 11935163

[23] Guichelaar MM Kendall R Malinchoc M Hay JE. Bone mineral density before and after OLT: long-term follow-up and predictive factors. Liver Transpl. (2006) 12:1390–402. doi: 10.1002/(ISSN)1527-6473 16933236

[24] Mart i G Gomez R Jodar E Loinaz C Moreno E Hawkins E. Long-term follow-up of bone mass after orthotopic liver transplantation: effect of steroid withdrawal from the immunosuppressive regimen. Osteoporos Int. (2002) 13:147–50. doi: 10.1007/s001980200006 11905524

[25] Monegal A Navasa M Guanabens N Peris P Pons F Martinez de Osaba MJ. Bone disease after liver transplantation: a long-term prospective study of bone mass changes, hormonal status and histomorphometric characteristics. Osteoporos Int. (2001) 12:484–9. doi: 10.1007/s001980170094 11446565

[26] Nightingale S McEwan-Jackson FD Hawker GA Macarthur C Khambalia AZ Lo L. Corticosteroid exposure not associated with long-term bone mineral density in pediatric liver transplantation. J Pediatr Gastroenterol Nutr. (2011) 53:326–32. doi: 10.1097/MPG.0b013e3182258656 21629126

