# Supplementary material for: Five-year evaluation of bone health in liver transplant patients: developing a risk score for predicting bone fragility progression beyond the first year
Source: Front Endocrinol (Lausanne). 2025 Feb 20;16:1467825. doi: 10.3389/fendo.2025.1467825 (PMC11882866; doi:10.3389/fendo.2025.1467825)
Supplement: Supplementary file 1 [file Table1.docx]

**Supplemental Table 1.** Proportion of patients with BMD progression within 18 months after the 1 year post-liver transplant follow-up (~2.5 years after transplant) according to Simplified Score in subset of patients with one or more follow-up visits after 1 year post liver transplant follow-up

| Simplified Risk Score | Fraction (%) with BMD Progression | 95% CI |
| --- | --- | --- |
| 0-2* | 2/41 (4.9%) | 0.6% to 16.5% |
| 3 | 1/58 (1.7%) | 0.0% to 9.2% |
| 4 | 2/53 (3.8%) | 0.5% to 13.0% |
| 5 | 6/39 (15.4%) | 5.9% to 30.5% |
| 6-8* | 9/22 (40.9%) | 20.7% to 63.6% |
| Scores observed by fewer than 20 patients were combined with the closest score. | | |
